# Carl Arthur Kollmann: Urologe, Venerologe und Puppenspieler in Leipzig

**DOI:** 10.1007/s00120-023-02163-9

**Published:** 2023-08-15

**Authors:** Friedrich H. Moll

**Affiliations:** 1 Institut für Geschichte, Theorie und Ethik der Medizin, Heinrich-Heine- Universität, Düsseldorf, Centre for Health and Society, Düsseldorf, Deutschland; 2 Urologische Klinik, Kliniken der Stadt Köln gGmbH, Neufelder Straße 32, 51067 Köln, Deutschland; 3 Deutsche Gesellschaft für Urologie e. V., Düsseldorf-Berlin, Düsseldorf, Deutschland

**Keywords:** Erinnerungskultur der Urologie, Fachentwicklung, Venero-Urologie, Geschichte der Medizin, Kulturgeschichte des Puppenspiels, Culture of remembrance in urology, Uro-venerology, History of medicine, History of science, Cultural history of puppet shows

## Abstract

Während die Erinnerungskultur zu dem aus Dresden stammenden sächsischen Urologen Felix Martin Oberländer innerhalb der deutschen Urologie gut entwickelt ist und sein Name in einem seit 1997 gestifteten Preis der Deutschen Gesellschaft für Urologie e. V. weiter fortlebt, ist die Erinnerung und das Wissen um Felix-Arthur Kollmann aus Leipzig in der Urologie verlorengegangen. Die Erinnerung an ihn in seinem weiteren akademischen Betätigungsfeld, dem Puppenspiel und der Puppenspielforschung ist bis heute lebendig.

## Abstract (en)

While Felix Martin Oberländer (born in Dresden, Saxony, Germany) is remembered in German-speaking urology and abroad, and his name has been honored since 1997 with an award named after him, the memory and knowledge of Arthur Kollmann of Leipzig (Saxony, Germany) seems to have been nearly forgotten within urology in Germany and abroad. However, the memory of him in other fields of science in which he was involved, e.g., puppets and puppetry-based research, remain vivid up to now.

„… trug Kollmann durch rege Mitarbeit, durch Einführung neuer Methoden und Instrumente viel zum weiteren Ausbau des vorliegenden Krankheitsbildes bei …“ [1]

„… er war auch sonst eine originelle Persönlichkeit …“^1^

## Zum Forschungsstand

Untersuchungen zu frühen, vor dem Jahre 1900 habilitierten Urologen stellen noch immer ein Desiderat der wissenschaftshistorischen Forschung in Urologie und Medizingeschichte dar. Das mag zum einen in den fachlichen Abgrenzungsproblemen zu dem sich im gleichen Zeitraum entwickelnden Fach Chirurgie liegen, zum anderen in der Fachgröße der Urologie selber. Im Jahre 1924 war die Urologie das kleinste medizinische Fach, für das ein Facharztstatus eingerichtet wurde.

Zu Arthur Kollmann existiert bisher nur ein Eintrag im Professorenkatalog der Universität Leipzig/Catalogus Professorium Lipsiensium, der vom Lehrstuhl für Neuere und Neueste Geschichte, Historisches Seminar der Universität Leipzig, online erstellt wurde und selber neben Universitätsquellen nur Kürschners Gelehrtenkalender aus dem Jahre 1931, der auf Eigenangaben beruht und seit dem Jahre 1925 in meist mehrjährigen Abständen erschien, berücksichtigt [2, 3]. In Isidor Fischers (1868–1938 Bristol) Standardwerk „Biographisches Lexikon der hervorragenden Ärzte der letzten fünfzig Jahre!“, Band 2, ist Arthur Kollmann ebenfalls vermerkt [4]. Das unterstreicht seine sichere Einbindung in den Fachdiskurs der Wissenschaften bis zu Beginn der 1930er-Jahre. Eine Ergo-Biobibliographie sowohl zum urologischen Oeuvre wie auch zum Forschungsfeld Puppenspiel und Zauberkunst existiert bisher nicht. Von Seiten der Kulturwissenschaften wurde er biographisch gewürdigt [5, 6]. In der sächsischen Biographie, einem Online-Projekt als „personengeschichtliches Lexikon zur Geschichte Sachsens“, ist der Name zwar registriert, jedoch ohne weitere Angaben [7].

## Biographische Skizze

Arthur Kollmann wurde am 8. Januar 1858 als Sohn des praktischen Arztes, Wundarztes und Geburtshelfers sowie Stadtverordneten Dr. med., Dr. phil. Carl Ferdinand Kollmann und dessen Ehefrau Anna Cäcilie Steeger (gest. 1922), Lessingstraße 5 in Leipzig, in ein arriviertes, gehobenes bürgerliches Elternhaus geboren.

**Figure 1:**
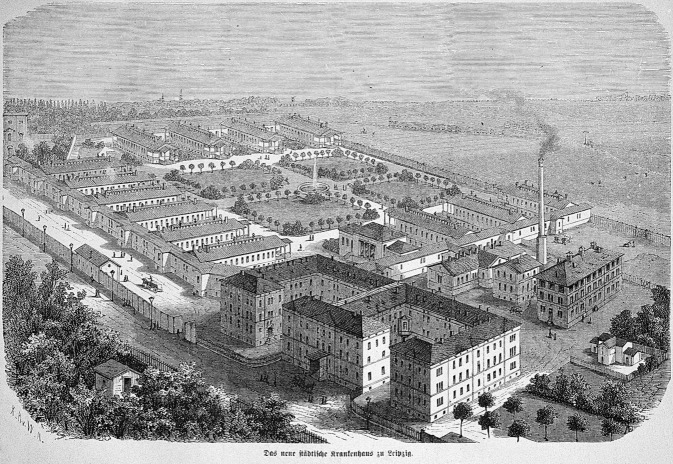

Seine Reifeprüfung legte er Ostern 1876 mit der mündlichen Prüfung am 23.–24. März am renommierten Leipziger Thomas Gymnasium (Thomas Schule/Schola Thomana) ab. Als Studienziel gab er zunächst Philosophie an [8]. Ab dem 29.04.1876^2^ studierte er zunächst im Sommersemester in Heidelberg und später ab dem Wintersemester an der Albertina in Leipzig das Fach Medizin. Die Medizinische Fakultät Leipzig zählte im letzten Drittel des 19. Jahrhunderts zu den bedeutendsten wissenschaftlichen Arbeits- und Unterrichtsstatten der Welt. An keiner vergleichbaren Einrichtung lehrte eine so große Zahl führender Fachwissenschaftler und anerkannter Gelehrter (C. Thiersch; [9–11]; Abb. 1).

Im WS 1881/1882 legte er die ärztliche Staatsprüfung nach vorangegangener Promotion (Ein Fall von primärem Schilddrüsenkarzinom) am 12. Februar 1881 ab.^3^

Hieran schloss sich eine Krankenbegleitung an die Riviera und Südfrankreich an. Dies war Ende des 19. Jahrhunderts eine sichere Möglichkeit, als junger, noch nicht länger niedergelassener Arzt über eine längere Zeit gesichert Geld zu verdienen.

Zur Vervollkommnung seins Fachwissens war Arthur Kollmann anschließend am Allgemeinen Krankenhaus der Stadt Wien (AKH) ab Herbst 1881 bis Ostern 1882 Volontärarzt auf der geburtshilflichen Station (Hofrath Josef Späth 1823–1896)^4^ und besuchte gleichzeitig Kurse in Dermatologie, Syphilis sowie Ohr- und Kehlkopferkrankungen. Seine venerologisch-urologische Ausbildung komplettierte er ab Februar 1882 in Paris, Hospital St. Louis, dem Hôpital Necker in Paris sowie dem Hôpital Midi Lorraine. Im Mai 1882 weilte er in London, Oxford und Cambridge.

Es ist davon auszugehen, dass er hier auch von seinem klinischen Lehrer Carl Wunderlich (1815–1877) beeinflusst worden war, der sich bereits im Jahre 1843 positiv über die in Paris stattfindende Spezialisierung medizinischer Fächer in einer Publikation geäußert hatte [15].

Zwischen 1882 und 1883 fasste er eine mikroskopische Studie unter dem späteren Dorpater Anatomen August Rauber (1841–1917) in Leipzig ab.^5^

Ab Mitte April 1884 war er Schiffsarzt beim Norddeutschen Lloyd und anschließend 3 Monate an den Zentren der US Medizin in Baltimore, New York, Philadelphia sowie Boston.

„*…* Sein Streben ging hier darauf, nicht nur im allgemeinen Land und Leute kennen zu lernen, sondern sich auch mit den medizinischen Lehranstalten und Universitäten unter besonderer Berücksichtigung seines speciellen Faches genau vertraut zu machen …“^6^

Im Februar 1885 war Kollmann wieder in Leipzig ansässig. Im Frühjahr 1886 legte er sein bezirksärztliche Examen ab und war Leichenschauarzt in Leipzig sowie ab 1887 Polizeiarzt bis zum Jahre 1925.^7^

„… Wir haben in Leipzig fast alle Klassen von Buhldirnen, wie sie in London, Paris, Berlin und anderen größeren Städten existieren, und das Geschäft der Lohnhurerei wird in allen Abstufungen von der größten Oeffentlichkeit bis zur groeßten Geheimhaltung betrieben ...“ [16, S. 24] – so stellte schon 1862 ein Zeitgenosse für die Prostitution fest und unterstrich hiermit die besondere Bedeutung der Polizeiarzttätigkeit nicht nur für die Stadt Leipzig und führt aus, dass „… die sanitätspolizeilichen Untersuchungen, wie man vorgegeben, den Zweck haben, einmal die Buhldirnen selbst, anderentheils die mit ihnen verkehrenden Männer vor Ansteckung zu wahren …“ [16, S. 24, 54] haben sollen.

„Die Polizeiärzte sind angewiesen mit größter Sorgfalt zu untersuchen, sie müssen sich daher des Mutterspiegels bedienen, müssen dies Instrument nach jeder Untersuchung selbst reinigen, müssen bei widerwärtigen und schmutzigen Frauenzimmern den Schlamm der Grube wegwischen und abtupfen, um der Behörde die Nachricht zu geben, der Grund der Grube sei in Ordnung …“ [16, S. 24, 60–61].

Parallel hierzu unterhielt er eine freie Praxis, in der er sich wohl zunehmend auf das sich entwickelnde Fach Urologie/Venerologie im Großstadtbereich spezialisierte. In diesem Zusammenhang bezeichnet ihn der 7 Jahre ältere Felix Martin Oberländer aus Dresden als seinen ältesten Schüler [17]. Dies lässt darauf schließen, dass beide Protagonisten der sächsischen Urologenschule in einem regen wissenschaftlichen Austausch über lange Zeit standen. Diese Mischung aus staatlicher Anstellung als Bezirks- und Polizeiarzt sowie freier Praxistätigkeit sicherte Arthur Kollmann ein regelhaftes, dem Lebensstandard gemäßes Einkommen und ermöglichte ihm zudem seine wissenschaftliche und große sammlerische Tätigkeit.

Im Jahre 1890 (1. August) habilitierte er sich und war damit im Deutschen Reich (Königreich Sachsen) der erste, der unter dem neuen Rubrum „Erkrankungen der Harnorgane“ die Venia legendi erhielt. Die im Vorjahr in Berlin habilitierten Urologen Max Nitze (1848–1906) und Carl Posner (1854–1928) waren an der Friedrich-Wilhelms Universität unter der formalen Fachspezifikation „Chirurgie“ bzw. „Innere Medizin“ habilitiert worden. Nitze hatte hierzu sein „Handbuch der Kystoskopie“ als Qualifikationsschrift eingereicht [18, 19]. Dies veranschaulicht deutlich den schwierigen Prozess der Fachetablierung an den jeweiligen Hochschulstandorten.

Somit war die sächsische Universität Leipzig, vielleicht auch aufgrund einer geographischen und hochschulpolitischen Nähe zu Wien, deutlich früher in der Zuteilung dieser neuen fachlichen Spezialisierung im Rahmen einer Venia legendi.

**Figure 2:**
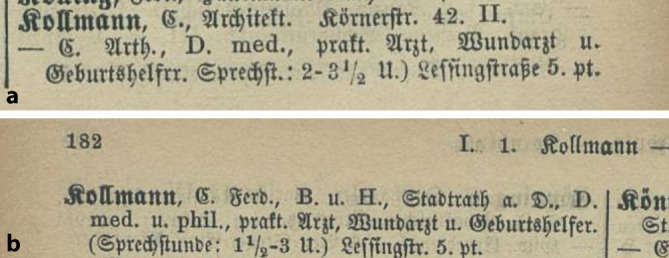

Während Kollmanns Qualifikationsschrift ein hämatologisches Thema beinhaltete, war Arthur Kollmanns Antrittsvorlesung „Die neueren physikalischen diagnostischen Methoden bei Erkrankungen der Blase und Harnröhre“ seinem eigentlichen urologischen Arbeitsfeld gewidmet. Er wurde im Jahre 1901 a. o. Professor in Leipzig. Noch zum Zeitpunkt seiner Habilitation war er unter seiner elterlichen Adresse Lessingstraße 5 gemeldet (Abb. 2 und 3; Tab. 1).

**Figure 3:**
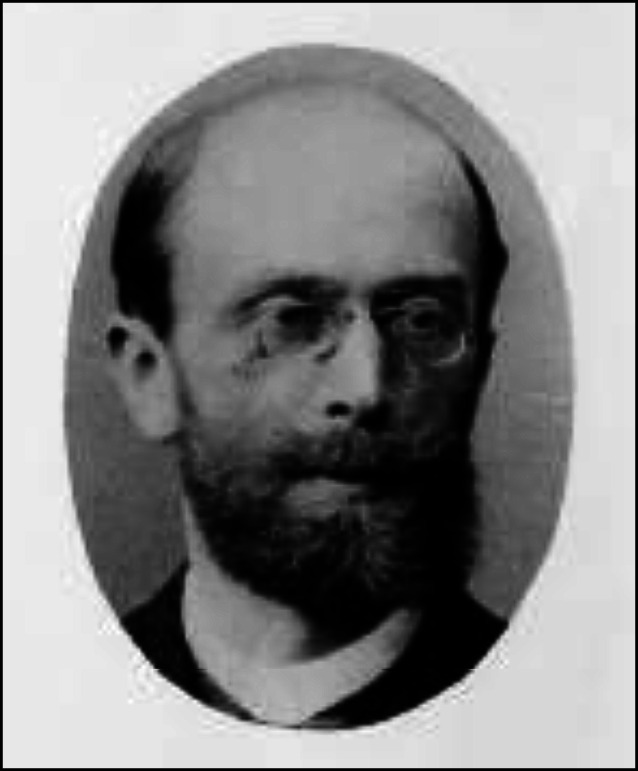

**Table 1:** 

1851 v. Ivanchich	Wien	Harnorgane
1872 Ultzmann	Wien	Erkrankungen Harnorgane
1874 Edler von Lavandal	Wien	Chirurgie der Harn- und Geschlechtsorgane
*1889 Nitze*	*Berlin*	*(Chirurgie)*
*1889 Posner*	*Berlin*	*(Innere Medizin)*
1890 Kollmann	Leipzig	Erkrankungen Harnorgane
1914 Kielleuthner	München	Urologie

Erst im Jahre 1892, im Alter von 33 Jahren, heiratete er Valeska Lietzmann (1864–1938) mit der er drei Töchter hatte [20]. Er wohnte in der Montbéstraße in Leipzig-Gohlis. Arthur Kollmann starb in Leipzig im Jahre 1941 im Alter von 83 Jahren und wurde auf dem Leipziger Nordfriedhof begraben [21].^8^ Im Jahre 1903 war ihm der Königlich-Sächsische Albrechts-Orden für geleistete Dienste im Staat, Wissenschaft und Kunst sowie „für gute bürgerliche Tugenden“ verliehen worden.^9^

**Figure 4:**
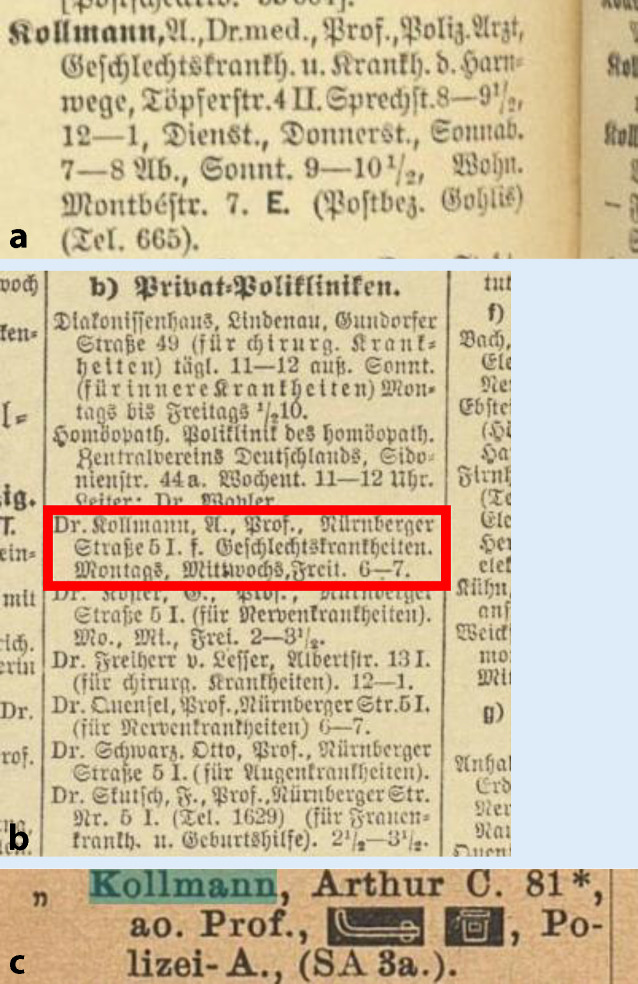

Als Leiter einer privaten Poliklinik für Hautkrankheiten in der Leipziger Nürnberger Straße prägte er den 8 Jahre jüngeren Urologen und Sexualmediziner Herrmann Rohleder (1866–1934) wesentlich [22]. Ein weiterer Schüler war Hans Wossidlo (1854–1918) [23]. Hier hielt er seit 1904 auch Teile seiner Vorlesungen an der Leipziger Universität „Hautkrankheiten, Syphilis und Krankheiten der Harnorgane“ ([24]; Abb. 4a–c und 5).

**Figure 5:**
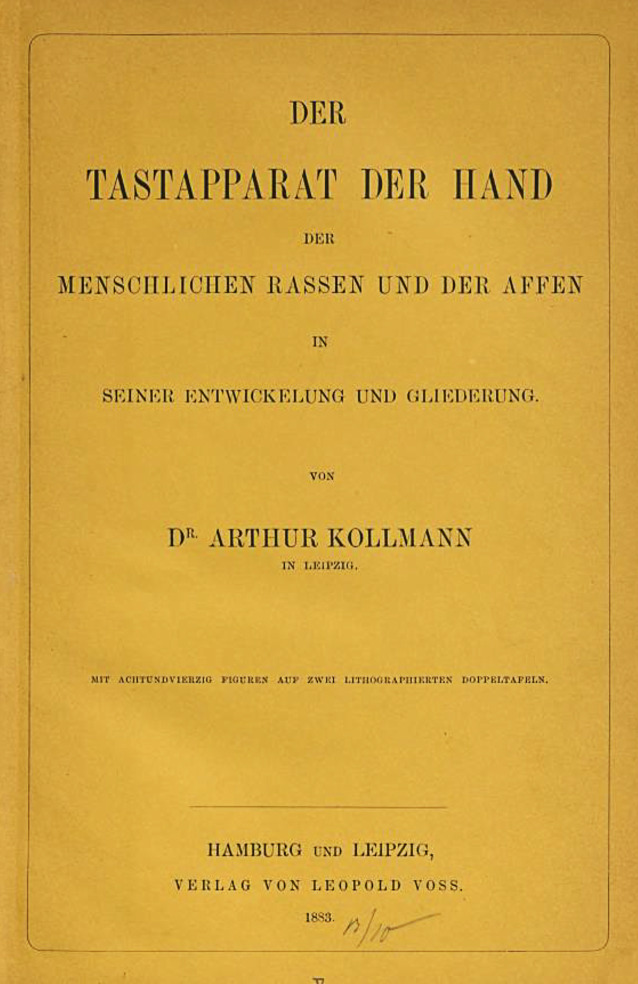

Dieser Lebensweg ist vor der Folie einer sich sehr rasch entwickelnden Industriemetropolemit Messestandort zu sehen. Seit 1870 war Leipzig mit 100.000 Einwohnern eine Großstadt des Deutschen Reichs. 1895 zählte die Stadt bereits 399.963 Einwohner, 1900 waren es 456.124 und 1905 konnten 503.672 Einwohnern personenregistriert werden [25]. Der enorme Bevölkerungszuwachs innerhalb kurzer Zeit resultierte einerseits aus der schrittweisen Eingemeindung der Vororte; Hauptursache für den rasanten Aufschwung der Stadt war der konsequente Ausbau zur Messemetropole. Anders als in anderen deutschen Städten verdrängte die moderne Mustermesse hier die bisher vorherrschende traditionelle Warenmesse innerhalb kurzer Zeit [26].

## Wissenschaftliches Oeuvre

Arthur Kollmanns erste wissenschaftliche Arbeit „Der Tastapparat der Hand der menschlichen Rassen und der Affen in seiner Entwickelung und Gliederung“ erschien 1883 bei Voss in Hamburg ([27]; Abb. 5).

Hieran schloss sich eine Arbeit über den Tastapparat des Fußes im Archiv für Medizin an [28].

In 1886 erschien dann noch die Übersetzung aus seiner Feder von Emanuel Edward Kleins (1844–1925; [29]) „Grundzüge der Histologie“ (Elements of Histology; [30]).

**Figure 6:**
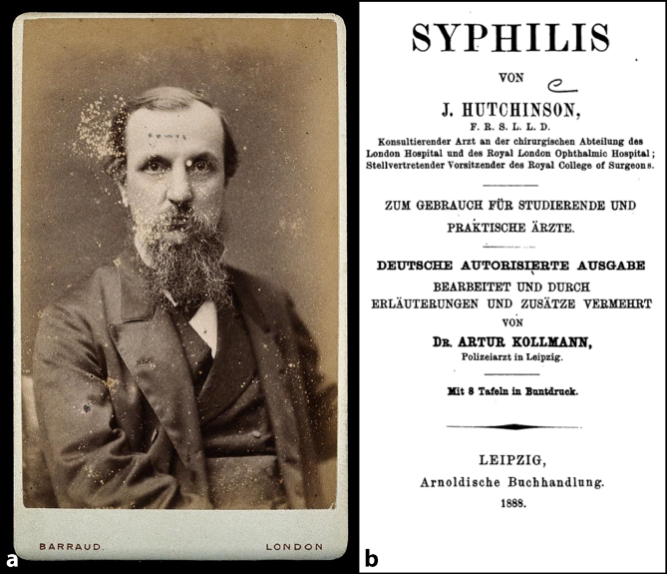

Im Jahre 1887 hatte sich Arthur Kollmann in dem Bereich Venero-Urologie bereits so etabliert, dass er das international bekannte und mehrfach in Europa und den USA aufgelegte Lehrbuch von Jonathan Hutchinson (1828–1913) ins Deutsche übersetzte [31–33]. Dieser war nicht nur wegen der Hutchinson-Trias eponymbildend geworden, sondern hatte auch durch die fälschliche Annahme, die vorsorgliche Beschneidung im Kindesalter könne beispielsweise die Erkrankungsrate an Syphilis um bis zu 49 % herabsetzen, das venero-urologische Fachwissen lange beherrscht, wie auch durch die Aufnahme der These, eine Beschneidung wirke der Masturbation entgegen (Abb. 6).

Auf nicht urologischem Gebiete ist noch Kollmanns Arbeit „Mikroskopische Blutbefunde bei Influenzakranken“, die in der renommierten *Berliner Klinischen Wochenschrift* erschien, zu erwähnen [34]. Diese gibt im wesentliche seine von Felix von Birch-Hirschfeld (1842–1899), Pathologe und Heinrich Curschmann (1846–1910), Internist, begutachtete Habilitationsschrift wieder.

**Figure 7:**
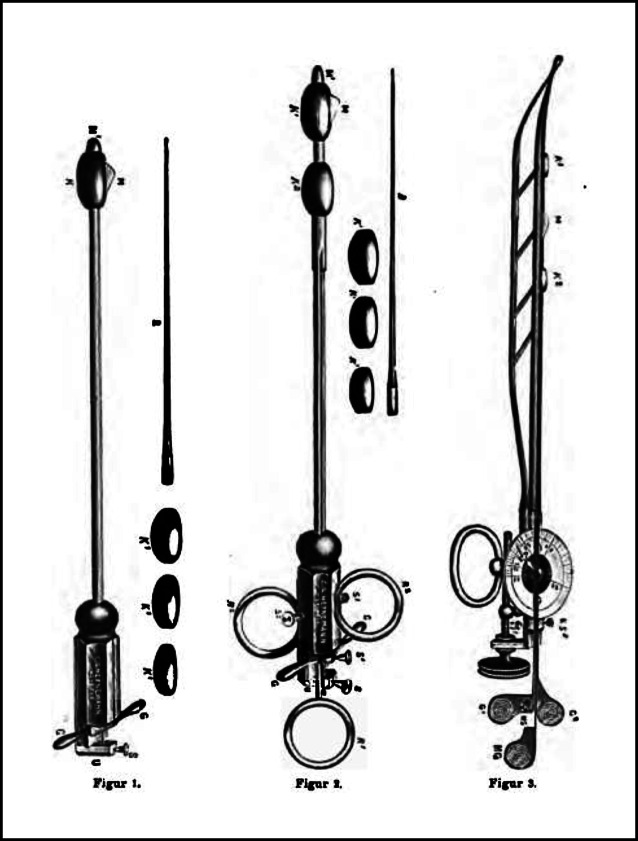

In den Jahren bis zur Ernennung zum a. o. Professor verfasste er eine Reihe von Einzelarbeiten mit urologisch-venerologischer Themenstellung, wobei er auch die Gerätemodifikationen zum Nitze-Oberländer-Urethroskop sowie Urethrotome und Zystoskopmodifikationen angab ([35–45]; Abb. 7).

**Figure 8:**
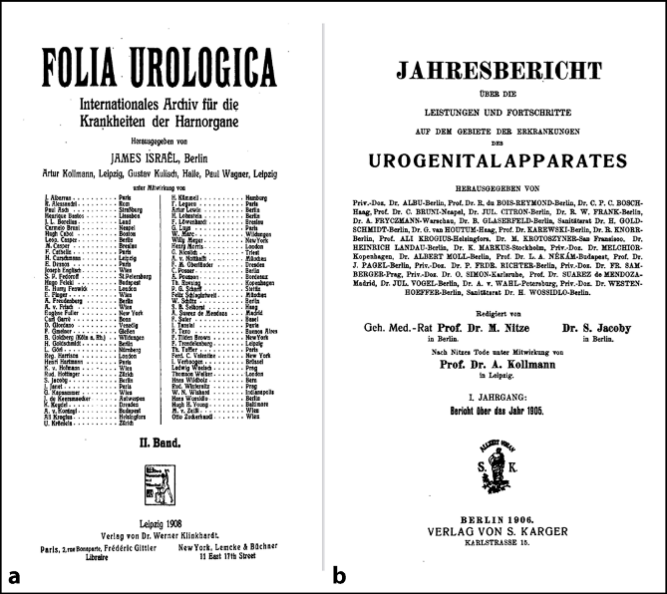

In den mit dem Nitze Schüler Samuel Jacoby (1867–1915), Berlin, herausgegebenen „Jahresberichte über die Leistungen und Fortschritte auf dem Gebiet der Erkrankungen des Urogenitalapparates“ Jahrgang 1–8 und den berühmten „Folia Urologica“ gehörte Arthur Kollmann prominent dem Herausgebergremium dieser Fachzeitschriften an. Dies unterstreicht, dass der arrivierte Wissenschaftler zwischen 1890–1920 als Vertreter der sächsischen Urologenschule auch in der Literatur fachprägend war und sein Themengebiet, die Behandlung der (chronischen) Gonorrhö, half, das Fachgebiet der Urologie zu konstituieren und fest im Wissenskanon der Kollegen zu verankern. Gleichzeitig kann dies sicherlich als ein Grund dafür angesehen werden, dass sein Name mit der Zunahme der klinischen Bedeutung und Abgrenzung der operativen Urologie zur konservativ betriebenen Venero-Urologie ab den 1920er-Jahren (*Zeitschrift für Urologische Chirurgie* ab 1913 bei Julius Springer, Handbuch der Urologie A. v. Lichtenberg, F. Voelcker, H. Wildbolz, 1926–1929 bei Julius Springer) sowie dem stattfindenden Generationswechsel im Fach selber vielfach in Vergessenheit geriet. Im Handbuch der Urologie hatte Kollmann kein entsprechendes Kapitel mehr bearbeitet, sondern dieses war netzwerkbedingt – wahrscheinlich durch Alexander von Lichtenberg (1880–1949) und Friedrich Voelker (1872–1955) als Herausgeber veranlasst – an den Berliner Urologen Arthur Lewin (1866–1939) vergeben worden. Arthur Lewin war gleichzeitig Schriftführer der alten DGfU. Dieser zitiert den Altmeister in seinem Beitrag ausführlich ([46]; Abb. 8).

Kollmanns wichtigste Buchpublikationen war zusammen mit dem nicht unumstrittenen Albrecht Freiherr von Notthafft, München (1868–1950; [47]) in der Reihe „Prophylaxe“ [48] der Band „Die Prophylaxe bei Krankheiten der Harnwege und des Geschlechtsapparates (des Mannes)“. Von Notthafft war für diese interdisziplinäre Gruppe besonders durch sein um die Wende zum 20. Jahrhundert erschienenes, mehrfach aufgelegtes „Taschenbuch der Untersuchungsmethoden für Dermatologen und Urologen“ bekannt gewesen.

**Figure 9:**
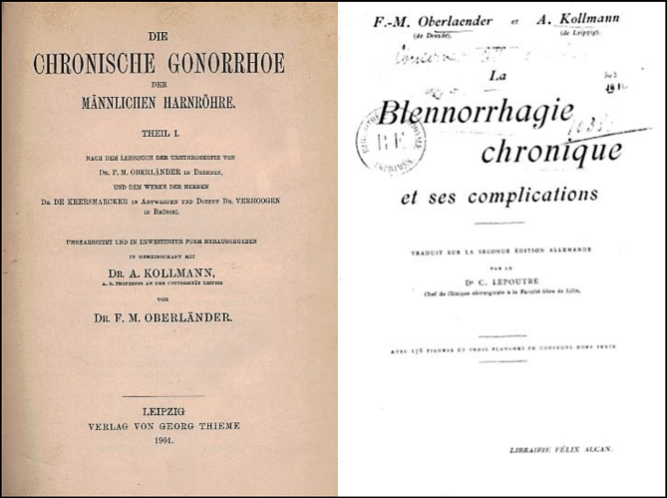

Mit dem renommierten Dresdener Vertreter der Urologie Felix Martin Oberländer (1851–1915; [49]) publizierte Kollmann das Standardwerk der Uro-Venerologie über mehr als eine Dekade „Die chronische Gonorrhoe der männlichen Harnröhre und ihre Komplikationen“ bei dem renommierten Leipziger Verlagshaus für Medizin, Georg Thieme, 1901/1905 verlegt, dem eine zweite Auflage im Jahre 1910 folgte ([50]; Abb. 9).

Die Publikation und die Autoren wurden von der französischsprachigen und auch der englischen Literatur breit rezipiert [51–53].

**Figure 10:**
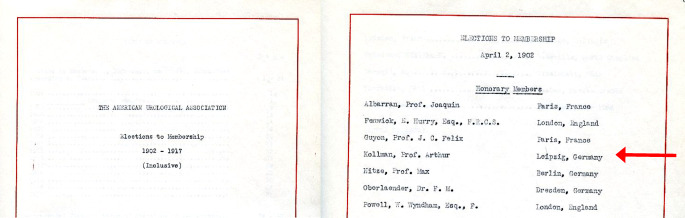

Arthur Kollmanns internationaler Bekanntheitsgrad führte zur Verleihung der Ehrenmitgliedschaft in der neu gegründeten American Urological Association im Jahre 1902, was auch den besonderen Status der sächsischen Urologenschule unterstreicht, da nicht nur Berlin und Wien neben London und Paris als europäische Zentren der Spezialfachentwicklung in den USA wahrgenommen wurden (Abb. 10). War er auch Gründungsmitglied der DGU im Jahre 1906/1907, so gehörte er im Gegensatz zu Felix Martin Oberländer keinem Leitungsgremium an [54].

Für das Handbuch der Haut- und Geschlechtskrankheiten von Josef Jodassohn (1864–1936 Zürich) bearbeitete Arthur Kollmann 1930 das technische Kapitel Urethroskopie [55]. Auch hier kann man den beginnenden Abgrenzungsprozess der urologischen Venerologie zur operativen Krankenhausurologie daran ablesen, dass Hans Boeminghaus (1893–1879), zu dieser Zeit in Marburg, das Kapitel „Die Chirurgie der Gonorrhoe“ verfasste in Anlehnung an ein Kapitel von Alexander von Lichtenberg (1880–1949). Dieser hatte im Jahre 1929 „Die Chirurgie der Gonorrhöe“ für das „Lehrbuch der Gonorrhöe“ von Abraham Buschke (1868–1943 Theresienstadt) und Erich Langer (1891–1957) [56], das ebenfalls bei Julius Springer in Berlin 1929 erschienen war, verfasst [57, 58].

**Figure 11:**
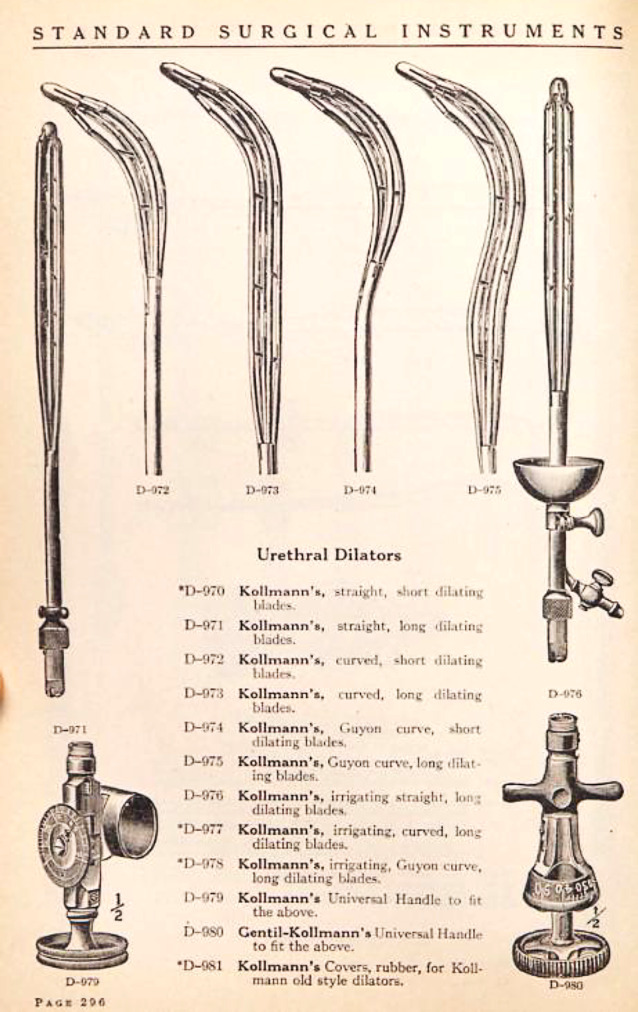

**Figure 12:**
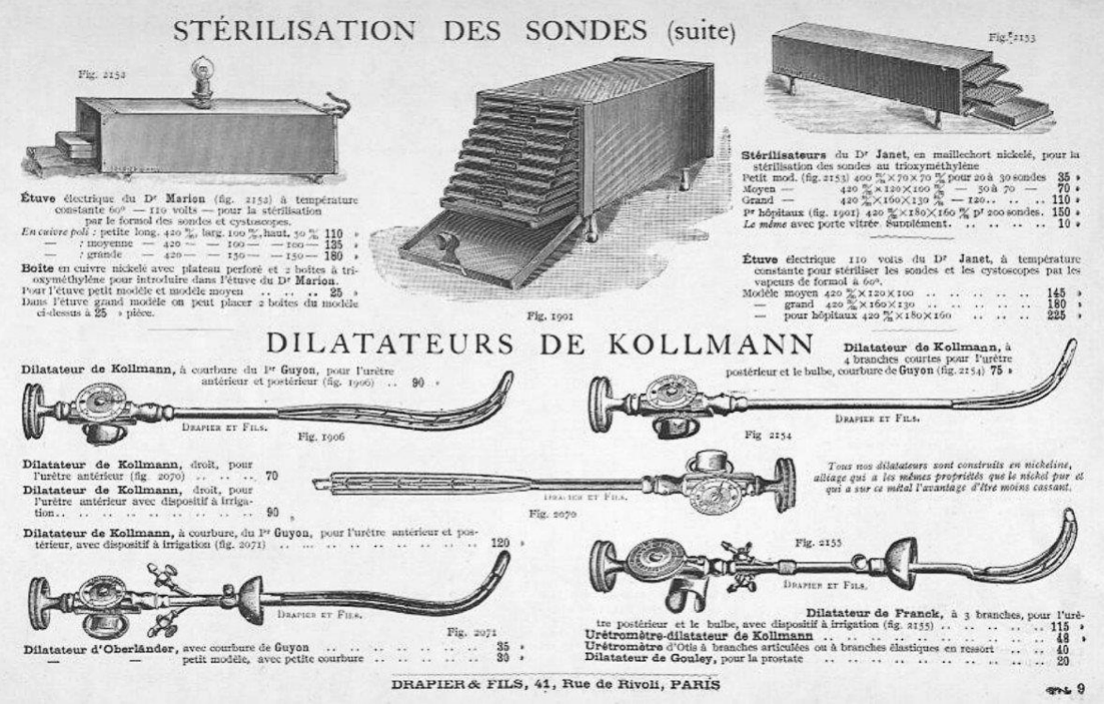

Die von Arthur Kollmann mit dem Leipziger Instrumentenbauer C. G. Heynemann (1857–1923), Elsterstraße 13 (gegr. 1890), entwickelten vierbranchigen Dilatatoren [59] in Krümmungen nach verschiedenen Autoren von Harnröhrenkathetern (u. a. Felix Guyon, Leopold von Dittel usw. [in Unterscheidung zu den Oberländer Instrumenten mit 2 Branchen]) waren für lange Zeit eines der führenden und weit verbreitetsten Therapieinstrumente bei der Dilationsbehandlung der chronischen Gonorrhoe in der vorantibiotischen Ära [60]. Diese sind heute immer noch zu erwerben ([61, 62]; Abb. 11, 12, 13 und 14).

**Figure 13:**
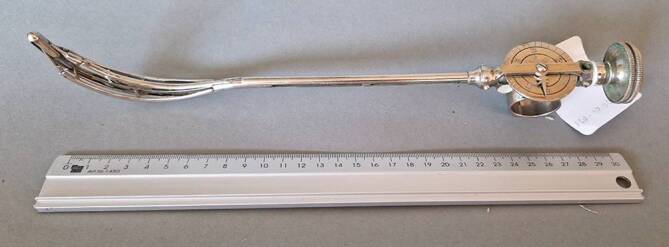

**Figure 14:**
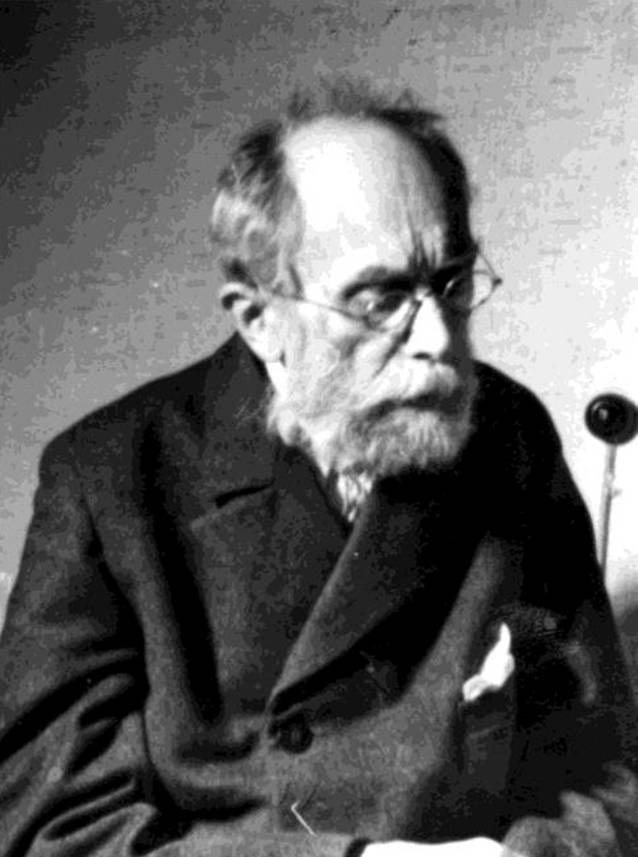

## Künstlerisches Oeuvre – Puppenspiel und Zauberkunst

Bereits in seiner Kindheit und Jugend war Arthur Kollmann mit dem Marionettentheaterprinzipal Carl Kapphahn (1823–1899) in Kontakt gekommen. Hieraus entwickelte sich ab 1895 eine private Sammlung, die sich als Förderung und Tradierung der kulturellen Eigenart dieser Kunstform verstand.^10^ Die hierbei entstandene Bibliothek ist Bestandteil der sächsischen Puppentheatersammlung heute in Dresden. Die Puppenspielliteratur (Textbücher, Akzidenzdrucke) sowie Titel zur Geschichte des Puppenspiels bildeten den Hauptbestandteil der Sammlung. Die Sammlung wurde ergänzt durch Ankäufe bei sächsischen Marionetten- und Handpuppenspielern. Seit 1959 ist sie im Radebeuler Hohenhaus untergebracht. Die ursprünglich dem Leipziger Völkerkundemuseum übereigneten Bestände aus den Sammlungen Kollmann und Lenn wurden, soweit sie den Bereich Puppenspiel betrafen, 1971 und 1993 der Dresdner Puppentheatersammlung hinzugefügt.

Arthur Kollmann gab sächsischen Puppenspielern Sachbeihilfen in Notlagen ab den 1890er-Jahren und erwarb Theatermaterialien wie Textbücher, Theaterzettel, Puppen, Bühnen und Fotografien. Seine wissenschaftlichen Erkenntnisse hierzu gab er in Vorlesungen am Ethnologisch-Anthropologischen Seminar der Universität Leipzig im Museum für Völkerkunde (Grassi-Museum) weiter. Kollmann publizierte hierzu vielfältig und wird auf diesem Gebiete bis heute wissenschaftlich rezipiert [63, 64], u. a. in der Zeitschrift *Der Bund*, Bern 1913 „Figurentheater und Schattenspiele“ [65] und gab auch ein Handbuch heraus [66]. Er organisierte auch in Leipzig Aufführungen mit dem Schwiegersohn Capphahns Georg Grube (1874–1943) u. a. Faust, ein Stück, das in der Capphahn’schen Aufführungspraxis eine längere Tradition besaß [67–70]. Kollmanns Sammlung aus Theaterzetteln, Theatertexten, Figuren, Proszenien und Dekorationen vermachte er initial dem „Leipziger Museum für Völkerkunde“, die diese später in die Dresdener Puppentheatersammlung/Museum für sächsische Volkskunst einordnete (Abb. 14). Einen wesentlichen Bestand an Theater- und Schaustellerzetteln hatte Kollmann über den Heidelberger großherzoglich badischer Hofantiquar Ernst Carlebach (1838–1923; [71]) 1895 aufgekauft. Diese stammten von dem Theaterdirektor Richard Kiesling (1810–1891) in Breslau, der diesen Bestand ab den 40er-Jahren des 19. Jahrhunderts angelegt hatte.

**Figure 15:**
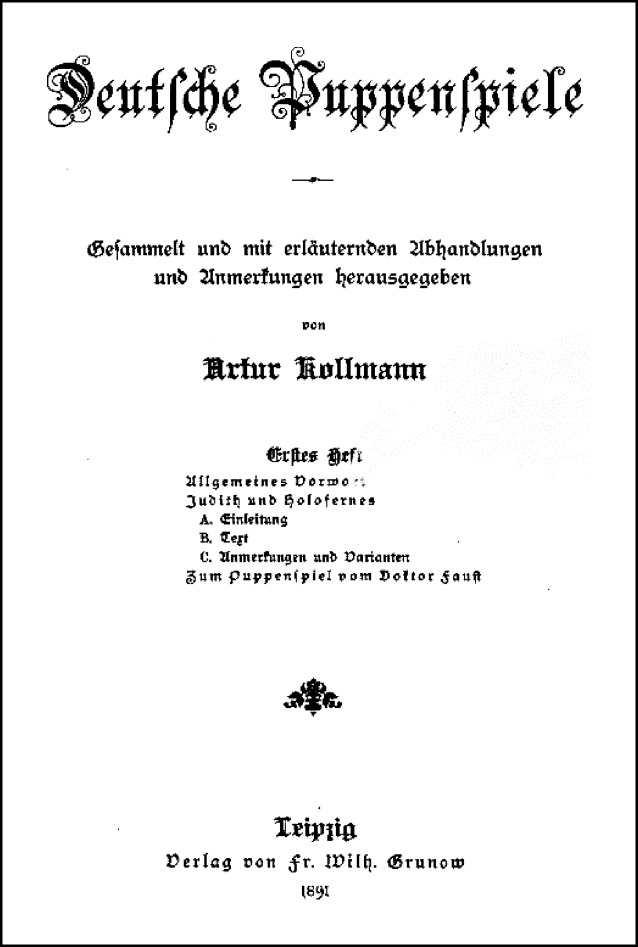

**Figure 16:**
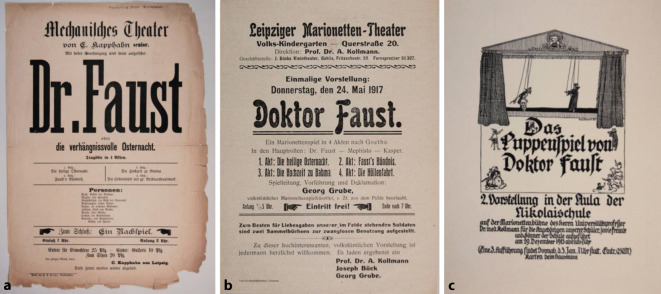

Ab 1918 arbeiteten der Lehrer und Puppenspiel Historiograph Otto Link (1888–1959) und Arthur Kollmann beim Aufbau der Sammlung eng zusammen [72]. Link sichtete und ordnete den Sammlungsbestand von Kollmann und erhielt hierdurch Zugang zum Kollmann’schen Schriftwechsel mit Puppenspielern in aller Welt [73–75]. Allein die Sammlertätigkeit und der Bestand zum Volksstück Dr. Faust ist aus literaturwissenschaftlicher Sicht beachtlich und wird in der entsprechenden Literatur rezipiert und gewürdigt ([76]; Abb. 15). Die Aachener Literaturhistorikerin Monika Fick hob vor kurzem in diesem Zusammenhang den Aspekt hervor, dass gerade Sammler- und Sammlungsbiographie(n) wie die Arthur Kollmanns für ein sozialgeschichtliches (Bildungsbürgertum und Bürgerengagement), für die nationalkulturellen, geschmackshistorischen und medienästhetischen Kontexte (und deren Wandel) ein Indiz seien sowie für den starken, individualistischen Persönlichkeitsbegriff des 19. Jahrhunderts ([77]; Abb. 16, 17 und 18).

**Figure 17:**
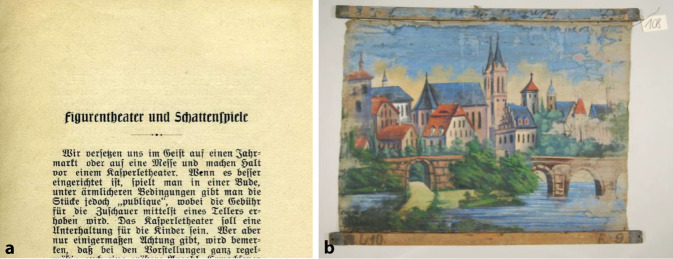

**Figure 18:**
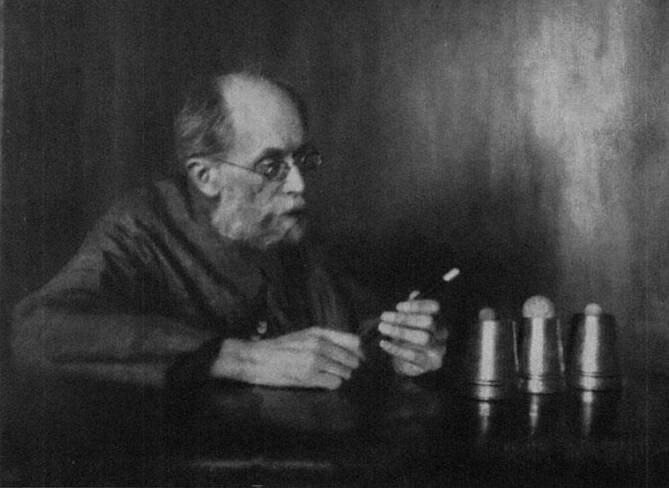

Sein Interesse an der Zauberei und magischen Requisiten und das hieraus resultierende umfangreiches Archiv zur Zauberkunde entwickelte sich wahrscheinlich aus der Beschäftigung Arthur Kollmanns mit dem Fauststoff. Er stellte es unter dem Titel „Sammlung zur Psychologie des Taschenspiels und er Wahrnehmungstäuschung zusammen“ und vermachte es dem „Magischen Zirkel von Deutschland“. Seit dem Zweiten Weltkrieg gilt die Sammlung jedoch als verschollen. Unter dem Pseudonym „Dr. Avon“ veröffentlichte Kollmann einige Artikel in der Zauberfachzeitschrift *Die Zauberwelt* [78–80].

In den 1920er-Jahren existierte an der Universität in Leipzig ein „Institut für Zauberkunde“, geleitet durch Arthur Kollmann. Kollmann beschäftigte sich besonders mit der psychologisch-medizinischen Wirkungsweise von Sinnestäuschungen auf den Menschen, hervorgerufen durch die Kunst des Zauberns ([81]; Abb. 18).

## Zusammenfassung – Fazit für die Praxis

Neben Felix Martin Oberländer in Dresden gehörte Arthur Kollmann in Leipzig zu den wichtigsten Vertretern der sächsischen Urologenschule, die neben der älteren Berliner Schule um Max Nitze (1848–1906), Paul Güterbrock (1844–1893) und Ernst Fürstenheim (1836–1904) die Fachspezialisierung in der Regel in eigener, niedergelassener Praxis, teils an Hochschulen assoziiert und habilitiert, im Großstadtbereich vehement vorantrieben und durch ihre jeweiligen lokalen, aber auch internationalen Netzwerke, zu den Nachbardisziplinen wie Chirurgie, Venero-Dermatologie und auch Frauenheilkunde maßgeblich prägten.

Die Vita dieses Urologen veranschaulicht, dass neben der tatkräftigen Förderung des Fachgebietes Urologie in einer renommierten Universitätsstadt nicht nur durch wissenschaftliche Beiträge, Entwicklung neuer Instrumente und Herausgeberschaften von Fachzeitschriften auch weitere Wissenszweige von Urologen auf akademischem Niveau betrieben wurden, die dann eine eigenständige Erinnerung weiter pflegen. Es wäre wünschenswert, wenn Arthur Kollmann in der Erinnerungskultur der deutschsprachigen und internationalen Urologie und deren geschichtswissenschaftlichen Diskurs wieder stärker fokussiert würde.

## Footnotes

1 Personalakte Universität Leipzig UAL PA 1458 pag 42 r.

2 „Arthur Kollmann studierte laut Matrikelband 1872/73–1894/95 unseres Universitätsarchives ab 29.04.1876 im Fach Philosophie“. Auskunft Jens Starick Universitätsarchiv Heidelberg vom 29.08.2022 per E‑Mail.

3 Personalakte Universität Leipzig UAL PA 1458.

4 In seiner aufsehenerregenden Inaugurationsrede „Das Studium der Medicin und die Frauen“ sprach er sich entschieden gegen das an der Universität Zürich schon seit 1864 gestattete Medizinstudium von Frauen aus. Zwar plädierte Späth für eine angemessene soziale Stellung und Würde der Frauen in der Gesellschaft, es fehle ihnen jedoch an der Befähigung für universitäre Studien (online: [12–14]).

5 Personalakte Universität Leipzig UAL PA 1458.

6 Personalakte Universität Leipzig UAL PA 1458 4.

7 Personalakte Universität Leipzig UAL PA 1458.

8 Personalakte Universität Leipzig UAL PA 1458 pag 40v.

9 Personalakte Verhandlungsbericht 1. Kongress Universität Leipzig UAL PA 1458.

10 Die späteren sächsischen Sammlung gingen wie die Buchbestände, die Puppenspieltextsammlung sowie das Konvolut der Theater- und Schaustellerzettel aus den Privatsammlungen des Leipziger Lehrers Otto Link (1888–1959), des Arztes Prof. Dr. Arthur Kollmann (1858–1941) und des Volkskundlers Dr. Alfred Lenn (1891–1955) hervor. 1952 schenkte Otto Link seine Sammlung zu Geschichte, Wesen und Erscheinungsformen des Puppenspiels dem sächsischen Staat.
